# Probiotics in Adolescent Prediabetes: A Pilot RCT on Glycemic Control and Intestinal Bacteriome

**DOI:** 10.3390/jcm8101743

**Published:** 2019-10-21

**Authors:** Charikleia Stefanaki, Athanasios Michos, George Mastorakos, Aimilia Mantzou, Georgios Landis, Paraskevi Zosi, Flora Bacopoulou

**Affiliations:** 1 Center for Adolescent Medicine and UNESCO Chair on Adolescent Health Care, First Department of Pediatrics, School of Medicine, National and Kapodistrian University of Athens, Aghia Sophia Children’s Hospital, 11527 Athens, Greece; georgioslandis@yahoo.co.uk (G.L.); fbacopoulou@med.uoa.gr (F.B.); 2 Department of Pediatrics, General Hospital of Piraeus ‘Aghios Panteleimon’, 18454 Piraeus, Greece; 3 Unit of Endocrinology, Diabetes mellitus, and Metabolism, School of Medicine, National and Kapodistrian University of Athens, Aretaieion Hospital, 11528 Athens, Greece; mastorakg@gmail.com; 4 Division of Infectious Diseases, First Department of Pediatrics, School of Medicine, National and Kapodistrian University of Athens, 11527 Athens, Greece; amichos@med.uoa.gr; 5 Unit of Clinical and Translational Research in Endocrinology, First Department of Pediatrics, School of Medicine, National and Kapodistrian University of Athens, Aghia Sophia Children’s Hospital, 11527 Athens, Greece; amantzou@med.uoa.gr

**Keywords:** Microbiome, prediabetes, hyperglycemic states, adolescence, probiotics

## Abstract

Dysbiosis of intestinal ecology could be implicated in prediabetes. The aim of this pilot randomized controlled trial (RCT) was to collect preliminary data on the effects of probiotic supplementation (Vivomixx©) on markers of glucose metabolism, intestinal microbiome composition, and intestinal health indices, of prediabetic adolescents. The intervention group was administered probiotic sachets twice daily for 4 months, while both intervention and control groups received weekly consultation sessions for a healthier lifestyle. Thirty-two participants were recruited (1.3 participants per month) and were randomized (16 in control and 16 in intervention group). Fifteen of them signed the inform consent and never entered the study (6 in control and 9 in intervention group). Thus, seventeen participants completed the study (10 in control and 7 in intervention group), with no serious adverse events. After the 4-month intervention, no difference was observed in the markers of glycemic control between the two groups, although a minor effect was observed for fasting glucose at 1-month, probably due to the initial higher adherence to the probiotic supplements. Modifications of the protocol procedures are warranted because of the high attrition rates and suboptimal compliance that were noted. Future studies and further RCTs with larger samples need to be conducted to fully elucidate the potential effects of probiotics in the glycemic control of prediabetic adolescents.

## 1. Introduction

The human intestinal microbiome seems to be associated with noncommunicable diseases, such as prediabetes, and it consists of the genomes of the bacteria (bacteriome), viruses (virome), and fungi (mycobiome) that form the composition of gut microflora. Molecular mimicry, epitope spread, and bystander effects in the immune system seem to mediate this association, leading to subclinical inflammation [1,2]. Oral administration of probiotics is of interest in the context of hyperglycemic states [3]. Probiotics may reduce the inflammatory response and concentrations of oxidative stress parameters, as well as increase the expression of adhesion proteins within the intestinal epithelium, thus reducing intestinal permeability, increasing insulin sensitivity, and reducing autoimmune response [4].

Prediabetes is a hyperglycemic state that precedes pathologic diabetic hyperglycemia. It is characterized by impaired metabolism of glucose and dysregulation of insulin excretion, manifested as elevated fasting glucose, impaired glucose tolerance, or a combination of both. Prediabetes is a pro-inflammatory state [5] with significant economic implications for health care systems globally [6]. 

In a recent review of the dysbiosis of the intestinal microflora in prediabetic patients, we summarized the evidence supporting decreased populations of intestinal bacteriome in prediabetic patients of all ages [7]. Studies implicate intestinal dysbiosis in hyperglycemic states, such as prediabetes. Few human studies have evaluated the impact of probiotics in prediabetic adults [8,9,10]. Kassaian et al. demonstrated beneficial effects of probiotics on the glycemic control of prediabetic adults [11], while promising results on glycemic control were also demonstrated in the study of Naito et al. when probiotics were administered in obese, prediabetic adults [12]. 

Adolescence is a stage of constant change in physical, sexual, emotional, and social characteristics due to hormonal actions, during which a physiologic decrease in insulin sensitivity occurs due to the increase in fat tissue mass and the growth hormone surge. The vulnerability of adolescents to insulin resistance states usually resolves by the completion of puberty. Yet, over the past two decades, the increase in the prevalence of obesity accompanied by alterations in the daily habits of adolescents have contributed to the rise of prediabetes in adolescence and of metabolic syndrome later in adult life, with prediabetes in adolescents acting like a precursor, also for type 2 diabetes mellitus [13,14,15,16].

The effects of probiotics on the glycemic control of adolescent prediabetic patients have not been thoroughly examined with randomized controlled trials (RCTs), where important practical issues, including funding, safety, and compliance, should be addressed. 

As pilot studies play a fundamental role in the development and design of definitive RCTs, we adopted an approach of a stepwise pilot RCT [17,18] with the primary aim to obtain preliminary data on the effect of a 4-month probiotic intervention on markers of glucose metabolism (serum glucose and hemoglobin A1c), intestinal microbiome composition (stool levels of microorganisms), and intestinal health indices (fecal pancreatic elastase, products of total protein breakdown, total fat, calprotectin, eosinophil protein X, secretory IgA) of prediabetic adolescents. 

## 2. Materials and Methods

### 2.1. Trial Design

This is a pilot randomized, controlled, two-armed, trial with a one-to-one allocation ratio. The basic protocol for the study has been described in detail elsewhere [19]. Feasibility was assessed by the following predetermined criteria: (a) recruitment rate of 1 participant per month; (b) five completed interventions for a total of 12 to 25 recruited participants with no serious adverse events for 24 months. The study was conducted at the tertiary Center for Adolescent Medicine and UNESCO Chair on Adolescent Health Care of the First Department of Pediatrics, National and Kapodistrian University of Athens, in Athens, Greece (‘study site’) from June 2015 until June 2017. The study protocol was approved by the Ethics Committee of the Aghia Sophia Children’s Hospital (repository number: 28931/11.02.2015) and was in accordance with the Helsinki Declaration for Human Studies. The trial was also registered at the Australian New Zealand Clinical Trial Registry Assigned number: ACTRN12615000470594, URL: https://www.anzctr.org.au/Trial/Registration/TrialReview.aspx?id = 364169 [19].

### 2.2. Participants—Procedures

Study participants comprised a convenience sample of male and female adolescents, who were screened for eligibility to be enrolled in the study over routine visits at the study site. Inclusion criteria: adolescents with Tanner stage III or IV for breasts or genitals for females or males, respectively; presence of prediabetes according to the American Diabetes Association (ADA) criteria: hemoglobin A1c (HbA1c) ranging from 5.7% to 6.4%, and/or fasting serum glucose concentrations ranging from 100 mg/dl to 125 mg/dl on two occasions (a,b), and/or serum glucose ranging from 140 mg/dl to 199 mg/dl at 2 h following an oral glucose tolerance test (OGTT) [20]; residence in the prefecture of Attika for at least 4 months following study entry, and willingness to participate in the study [19]. 

Exclusion criteria: short-bowel syndrome; current hospitalization; co-morbid infection; co-morbid hereditary and/or acquired immunodeficiency; genetic defects of insulin action, diseases of the exocrine pancreas, gestational diabetes, endocrinopathies, diabetes; Stiffman’s syndrome; current or within the past month treatment with antibiotics; intake of yogurts and products containing probiotics or any immuno-modulating agent in the prior two months; history of diarrhea; seropositivity for the hepatitis B virus surface antigen (HBsAg), core antibody (HBcAb) and/or established diagnosis of any hepatic disorder; atopic dermatitis; epilepsy; established or possible pregnancy; chronic life-threatening disease, such as neoplasia and severe psychiatric illness [19].

After screening for eligibility, written informed consent was provided by the adolescents and their parents/legal guardians (for those younger than 18 years) before participation in the study. 

Upon study entry (baseline) and study termination (post-intervention), morning, fasting blood, and stool samples were collected on-site from each participant. At baseline and at the end of each month (1st, 2nd, 3rd, 4th month) during the intervention period, study participants visited the study site early in the morning after a 12-h fast, and blood samples were obtained for the assessment of fasting glucose concentrations [19]. At baseline and post-intervention, the measurement of HbA1c was also performed in the participants of both groups.

After the collection of the samples, the participants had a brief meeting with the principal investigator (C.S.) to report the number of sachets they had consumed during the previous month and any adverse reactions. Finally, they had a counseling session with a designated accredited dietician (G.L.) about a healthy lifestyle. The same procedure was followed for the control group. Dietary, probiotic supplementation was administered only to the intervention group while there was no placebo administration to the control group [19]. 

### 2.3. Interventions

Probiotic supplements were administered daily, post-prandially (one sachet after lunch and another after dinner) for 4 months. Each sachet of probiotics contained 450 × 10^9^ colony forming units (CFUs) of *Streptococcus thermophilus* (DSM24731), *Bifidobacteria breve* (DSM24732), *Bifidobacteria longum* (DSM2473), *Bifidobacteria infantis* (DSM24737), *Lactobacillus acidophilus* (DSM24735), *Lactobacillus plantarum* (DSM24730), *Lactobacillus paracasei* (DSM24733), *Lactobacillus delbreuckii* subspecies *bulgaricus* (DSM24734); available in the European Union as Vivomixx^®^ and in the United States of America as Visbiome^®^ [19].

Weekly counseling to promote a healthy lifestyle and restore intestinal ecology and glycemic homeostasis was provided to both adolescent groups. The role of the traditional Mediterranean diet was emphasized by a designated accredited dietitian (G.L.). General advice was given for high intake of extra virgin (cold pressed) olive oil, vegetables (including leafy green vegetables), fruits, cereals, nuts and pulses/legumes, moderate intake of fish, meat and dairy products and low intake of eggs [19,21]. The lifestyle intervention is unstandardized, and it was part of the usual follow-up for adolescents visiting the study site. 

Study participants were encouraged to perform moderate to vigorous exercise for a minimum of 30 min each day. Moderate to vigorous exercise was defined as the exercise causing “some increase in breathing and heart rate usually associated with brisk walking, dancing, swimming, or cycling on flat terrain”. In exercise physiology terms, the energy expended was at least 3 metabolic equivalents (METS) [19,22]. 

### 2.4. Outcomes

Participants’ fasting glycemia was evaluated 5 times during the 4-month study period: at baseline and at the end of the 1st, 2nd, 3rd, and 4th month. Blood samples were drawn from each participant at 08:00 a.m. by venipuncture after a 12-h overnight fast. Glycated hemoglobin HbA1c was immediately determined by the Siemens DCA Vantage point-of-care immunoassay analyzer (Siemens Health Care Diagnostics Ltd., Frimley, Camberley, UK) with intra- and inter-assay coefficients of variation (CVs) of 5% and 8%, respectively. Blood samples were centrifuged for 15 min at 2200 × *g* at 5 ℃, and the supernatant serum was analyzed for the determination of biochemical and hormonal parameters. Serum glucose concentrations were quantified by an automated analyzer system using available commercial colorimetric assay kits (glucose god/pap kit with intra- and inter-assay CVs of 5.7% and 7.8%, respectively). Serum insulin concentrations were measured with an electrochemiluminescence immunoassay using the automated analyzer Cobas e411 and the Elecsys Insulin Kit (Roche Diagnostics, Basel, CH; intra- and inter-assay CVs of 2.0% and 2.8%, respectively). [19].

Morning stool samples were collected on-site from each participant at baseline, and after the 4-month intervention. Products of (i) digestion and absorption: fecal pancreatic elastase in micrograms per gram; products of total protein breakdown in micromole per gram; total fecal fat (valerate, isobutyrate, isovalerate) in milligrams per gram; triglycerides in milligrams per gram; long-chain fatty acids (LCFA) in milligrams per gram; cholesterol in milligrams per gram; phospholipids in milligrams per gram, (ii) inflammation: calprotectin in micrograms per gram; eosinophil protein X (EPX) in micrograms per gram; fecal secretory IgA in micrograms per gram; and (iii) metabolism: short-chain fatty acids (SCFA) in micromole per gram; n-butyrate concentration in micromole per gram; n-butyrate percentage (%); acetate percentage (%); propionate percentage (%); beta-glucuronidase in Units/g, were evaluated before and after the intervention [19,23]. 

Semiquantitative determination of the levels of microorganisms in participants’ stool samples was performed with the use of 16s PCR and the incorporation of SYBR green for result determination using the GI Effects 2200 kit (GENOVA Diagnostics Inc. Asheville, NC 28803, USA), also described elsewhere [19]. The selection of the kit was performed according to the Human Microbiome Project results of human intestinal bacteriome composition [24].

### 2.5. Randomization—Blinding

An online randomization internet site (www.random.org) was used to assign study participants to intervention and control groups. Randomness was generated from the atmospheric noise. The random allocation sequence was implemented by a designated clinical assistant who was not otherwise associated with the trial. No blinding or concealment was used [19].

### 2.6. Statistical Methods

Statistical analysis was performed using the SPSS v.21 statistical software (IBM. Co., New York, NY, USA). The level of statistical significance was set at two-sided *P* < 0.05. Characteristics of the study sample were presented by group, in terms of median, Q25, Q50, and Q75 for quantitative variables, and absolute numbers and percentages for qualitative variables, due to the small sample. For group comparisons, the Mann–Whitney and Wilcoxon’s and Friedman’s tests for quantitative variables were used to evaluate statistically significant differences between the two groups and within the two groups before and after the intervention, or between multiple measurements, respectively, and χ^2^ tests or Fisher’s exact tests for qualitative variables were employed. Spearman’s correlation was performed to assess correlation between the variables. Data were analyzed using the intention-to-treat analysis. No stratification by age was employed due to the small number of study participants [17,19].

## 3. Results

### 3.1. Recruitment and Baseline Characteristics

**Figure 1:**
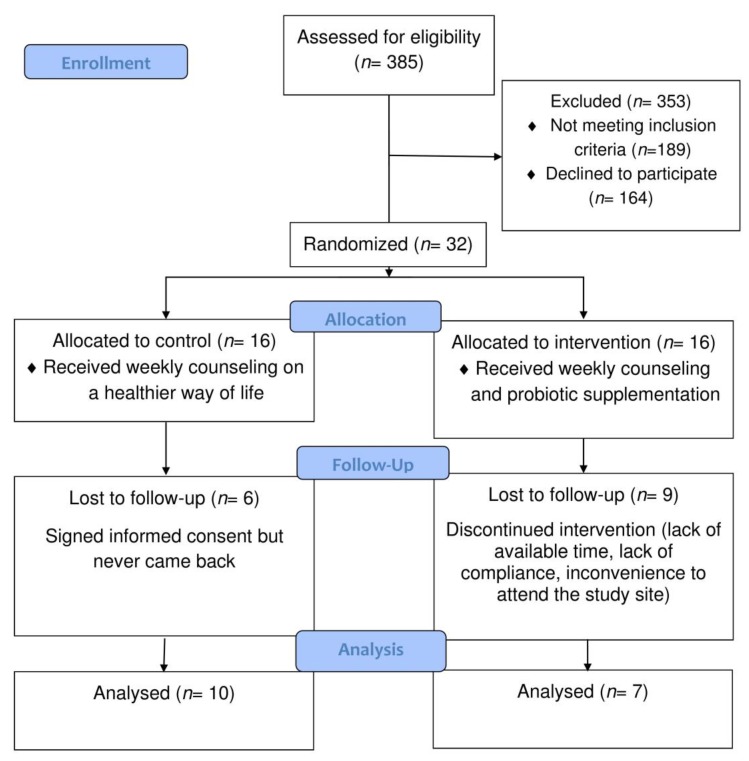
Study participants’ flow chart.

A total of 385 adolescents were initially screened for enrollment in the study. From them, 164 declined participation in the study, and 189 were excluded because they did not meet the inclusion criteria (150 did not meet the age range for inclusion, and 39 were diagnosed with a psychiatric disorder). Overall, 32 participants were recruited in 24 months (mean value = 1.3 participants per month). The 32 eligible adolescents for the study were randomized to control and intervention groups. Six and 9 participants of the control (attrition rate = 37.5%) and the intervention group (attrition rate = 56.25%), respectively, dropped-out and were subsequently excluded from the study. Finally, 17 adolescents (males, 5 out of 10 and 3 out of 7, in the control and the intervention group, respectively) were included in the study (Figure 1; Table 1). Study participants’ characteristics at enrollment are presented in Table 1. There were no statistically significant differences between the two groups for the majority of the baseline characteristics, except for the percentage of participants with a positive family history of diabetes that was significantly greater in the control as compared with that of the intervention group (*p* = 0.009). 

### 3.2. Outcomes and Estimations

#### 3.2.1. Between-Group Differences

After the first month of probiotic supplementation, fasting blood glucose concentrations were significantly lower in the intervention as compared to the control group (*p* = 0.043; Table 2). Glycemic control, intestinal immunity, and metabolic markers did not differ between the two groups after the 4-month intervention. 

At baseline, the excretion of total fecal fat, fecal LCFA, and phospholipids in the intervention group was lower than that of the control group (*p* = 0.03; *p* = 0.03, and *p* = 0.01, respectively; Table 2). Post-intervention, no statistically significant differences were detected in the concentrations of fecal fats, fecal LCFA, or phospholipids between the intervention and the control group (Table 2). 

Intestinal bacteriome analysis revealed statistically significant differences between the two groups; the intervention group showed significantly lower populations of the following bacteria: *Barnesiella* spp. (*p* = 0.01); *Butyrivibrio crossotus* (*p* = 0.01), *Faecalibacterium prausnitzii* (*p* = 0.01); *Collinsella aerofaciens* (*p* = 0.03); *Escherichia coli* (*p* = 0.01) and *Akkermansia muciniphila* (*p* = 0.03), when compared with the control group. 

#### 3.2.2. Within-Group Differences

(a) **Control Group**: At the end of the 4-month study period, fecal calprotectin concentrations were significantly decreased in the control group following the lifestyle intervention (Wilcoxon Z = −2.207; *p* = 0.03; Table 3). Statistically significant increases were noted in the populations of *Butyrivibrio crossotus* (Wilcoxon Z = −2.201; *p* = 0.03) and *Proteobacteria phylum* (Wilcoxon Z = −2.201; *p* = 0.03; Table 3) in the control group. (b) **Intervention Group**: Although, the required sachets of probiotics per participant for the 4-month intervention summed up to 240 sachets in total, the mean number of the received probiotic sachets was equal to 110 (Table 4). There was a statistically significant deviation from the expected amount of the probiotic supplementation (*t* (6) = −8.892; 95% C.I.: (−142.45) – (−80.97); *p* < 0.001). The intervention group demonstrated significantly lower post-intervention HbA1c (*p* = 0.03) and glucose (*p* = 0.03) concentrations, compared to their respective baseline values (Friedman’s test = 10.69; *p* = 0.03; Table 2). Post-intervention, no other statistically significant differences in the study variables were found in this group. 

### 3.3. Adverse Effects—Harms

The intervention group reported bloating, flatulence, and constipation, which constitute well-known adverse effects of probiotic supplementation [25,26]. These symptoms decreased with continued use as the participants of the intervention group reported a decrease in the frequency of their symptoms on a monthly basis. All participants of the intervention group scaled back on the probiotic dose due to these symptoms. No harm or serious adverse effects were noted. 

## 4. Discussion

We found glycemic control, intestinal immunity, and metabolic markers did not differ between the two groups at the end of the study period. However, after the first month of the study, fasting glucose concentrations were significantly lower in the intervention group as compared with those of the control group, probably due to the initial higher adherence to the probiotic supplementation. Afterward, this difference between the two groups disappeared, presumably due to the suboptimal compliance to probiotics of the participants in the intervention group. HbA1c values and fasting glucose concentrations were significantly lower than the baseline corresponding values when compared with their respective values, in the participants of the intervention group after the intervention period. 

In the past, in two randomized controlled trials, the administration of a combination of freeze-dried *Lactobacillus acidophilus*, *Bifidobacterium bifidum*, *Bifidobacterium lactis*, and *Bifidobacter longum* probiotic to prediabetic adults resulted in decreased fasting blood glucose concentrations [8,11,12], fasting insulin concentrations, HbA1c values, and insulin resistance indices [8,11]. 

The administration of probiotics may decrease bacterial lipopolysaccharides (LPSs) and pro-inflammatory cytokines, thus resulting in improvement of insulin sensitivity [27]. LPSs of intestinal Gram-negative bacteria increase pro-inflammatory cytokines, which are involved in the pathophysiology of insulin resistance [28]. In the intervention group of the present study, the majority of the bacteria populations that decreased following intervention were Gram-negative (*Barnesiella* spp., *Escherichia coli*, *Akkermancia muciniphila*). Possibly, the LPSs of these bacteria were involved in the process of insulin resistance [29,30]. The exact mechanism of the effect of probiotics on glycemic control is still under investigation. In contrast, no statistically significant differences were found among fasting glucose concentrations or HbA_1c_ values measured at the study time-points in the control group.

Furthermore, in the present study, at baseline, the participants in the intervention group exhibited significantly decreased fecal fat (free fatty acids, such as LCFA, and phospholipids) as compared to the participants of the control group, while at the end of the study this difference disappeared. The increase in free-fatty acids flux is implicated in the development of insulin resistance via the Randle cycle in the liver and muscles and through direct or indirect (derived from triglyceride deposits) generation of metabolites, which alter the insulin signaling pathway [31]. Alleviating the excess of free fatty acids is a target for the treatment of insulin resistance, especially in patients who exhibit impaired fasting glucose because they also demonstrate hepatic insulin resistance [32,33,34]. Lately, it has been demonstrated that non-alcoholic fatty liver disease (NAFLD) is directly implicated in the pathogenesis of hyperglycemic states [35].

A recent study showed that diets targeting NAFLD increase fecal fat excretion and bile acid conversion [36]. Interestingly, in the present study, fecal calprotectin concentrations of the control group decreased following lifestyle intervention alone, indicating a decrease in subclinical intestinal inflammation. The lifestyle intervention may have contributed to this reduction. No statistically significant differences were found between the intervention and the control groups regarding the markers of intestinal metabolism and those of immunity, probably due to the nature of this study and the small number of participants or the short duration of the intervention. 

At the end of the study, the intervention group demonstrated significantly lower populations of Barnesiella spp. and *Butyrivibrio crossotus, Collinsella aerofaciens, Faecalibacterium prausnitzii, Escherichia coli, Akkermancia muciniphila*, compared to the control group. In the past, populations of *Barnesiella* spp. were found increased in genetically-prone obese, prediabetic mice as well as in the presence of diet-induced diabetogenic intestinal environment [37], while *Butyrivibrio crossotus,* which has been implicated in the onset of insulin resistance [38], exhibits reduced capacity for uptake of branched-chain amino acids (BCAA; valine, leucine, and isoleucine). The latter promotes pro-inflammation in the intestine via activation of the mammalian-target for rapamycin complex (m-TORC) signaling and has been found increased locally in diabetic and prediabetic patients [38,39].

At the end of the study, the control group exhibited significantly greater populations of *Butyrivibrio crossotus* as compared to baseline. *Collinsella aerofaciens,* a bacterium well-known for its association with pro-inflammatory processes, is associated with the production of the pro-inflammatory cytokine interleukin-17A (IL-17A) and has been implicated in the alteration of intestinal permeability [40]. *Faecalibacterium prausnitzii* is a marker of intestinal health due to its ability to produce butyrate, which enhances anti-inflammation. Recently, it was demonstrated that strains of this bacterium, with different genomes, produce variable quantities of butyrate [41] in obese and lean diabetic patients [42]. Regarding *E. coli,* glucose is the most suitable substrate for its growth. Because intestine in prediabetic patients is rich in glucose, the growth of *E. coli* is favored [43]. The decrease in the population of this bacterium in this study should be corroborated with the improvement of glycemic control in the intervention group at the end of the study. Greater populations of *Proteobacteria* phylum were observed in the participants of the control group at the end of the study as compared with baseline populations. This phylum, including *E. coli*, is implicated in the pathogenesis of adult prediabetes [44].

Hyperglycemia reprograms intestinal epithelial cells via the upregulation of their GLUT-2 receptors resulting in intestinal barrier dysfunction and dysbiotic microbial influx via metaflammation [45]. These bacteria are implicated in dysbiosis and dysregulation of glycemic control, which, in the present study, improved following probiotic supplementation. The latter resulted in decreased populations of bacteria and the improvement of glycemic control. Thus, probiotic supplementation in prediabetic adolescents seems to have improved glycemic control via regulation of intestinal bacteriome, and therefore, via a decrease in intestinal metaflammation. On the other hand, weekly counseling on a healthier way of life led to a decrease in intestinal inflammation parameters in the control group, but it did not suffice for the improvement of glycemic control.

Last but not least, we observed significant attrition rates due to various barriers (lack of time, lack of self-management, and inconvenience in attending the study site) as well as lack of compliance with probiotic administration. More specifically, the majority of adolescents in the control group omitted to take the 2 daily doses, due to forgetting, disorganized time schedule, or inconvenience in carrying the sachets along when away from home. Others claimed embarrassment during the probiotic consumption, especially among peers, as they were reluctant to discuss their prediabetes within their social environment. The adolescents in the intervention group consumed about half of the recommended probiotic dose on a monthly basis. Many studies in the current medical literature have also evaluated the problem of impaired compliance to various treatments in this age [46,47].

It should be noted that the results of this pilot study should not be generalized to other probiotic formulations differing in the number of live/dead bacteria, strains type, or manufacturing processes when target populations are children or adolescents. Future studies need to address these barriers with counteracting facilitators. Facilitators to self-management may include a daily routine and daily cues to remembering to take the probiotics, weekly family psychologic consultations along with weekly dietary consultations about the beliefs regarding the probiotic supplementation, prediabetes, and the communication with family members and peers about prediabetes.

To our knowledge, this pilot study was the first to evaluate the effects of probiotic administration to prediabetic adolescents upon glycemic control and concurrently assess intestinal health, metabolic markers, and bacteriome composition. The results of the study support the absence of serious adverse effects but suboptimal adherence to probiotic intervention. Adolescents are a special target group that usually lacks self-management and self-efficacy and presents defiance to various treatments [48]. The findings of this study seem modestly encouraging with respect to probiotic supplementation in adolescents with prediabetes. The main limitations of this study remain the small number of participants and the suboptimal compliance to the probiotic consumption. However, our main aim was to obtain preliminary data, in the context of a pilot study, that could be used for future well-designed studies. The lack of standardized questionnaires about adherence to lifestyle counseling and probiotic supplementation, or details on adverse effects, is a potential unmeasured confounder. However, even if the possibility of selection bias was slight since the two groups did not differ significantly at baseline, due to the observed high attrition rates in the two groups, there seems to be a significant possibility of attrition bias. Finally, this was a small, but quality sample, as true prediabetes in adolescence is considered to be rare [13,49].

## 5. Conclusions

According to the results of this pilot trial, minor differences were revealed in the glycemic control status and altered homeostasis of the gut following a 4-month administration of a diverse mix of probiotics in the intervention group of overweight/obese prediabetic adolescents. Probiotics were relatively well-tolerated and seemed to moderately improve glycemic control and intestinal health. Modifications of the protocol procedures should be applied in future studies to improve adherence.

Any firm conclusion about the clinical use of probiotics in the amelioration of glucose control in prediabetic adolescents cannot be drawn. Future studies and further RCTs with larger samples, weekly meetings, and family psychologic counseling along with reminder systems are to be conducted to fully elucidate the potential effects of probiotics in the glucose control of prediabetic adolescents.

This study contributes to encouraging results to the current knowledge about the potential of probiotics as adjunct treatments of hyperglycemic states. Future studies of larger samples and more sophisticated design should address the compliance issues, particularly in children and adolescents.

## Figures and Tables

**Table 1:** Characteristics of the study sample.

Variable	Control Group *N* = 10 (Median-IQR or *n* (%))	Intervention Group *N* = 7 (Median-IQR or *n* (%))	*p* Statistical Significance Mann–Whitney Test
Age (in years)	13.50 (12–16.25)	15 (14–16)	*p* = 0.41
Body mass index (BMI) z-score			
Baseline	1.55 ((–0.65)–2.12)	2.2 (1.30–2.60)	*p* = 0.08
Post-intervention	1.30 ((−0.32)–2.12)	2.2 (1.50–2.60)	*p* =0.07
Male Gender	5 (50%)	3 (42.9%)	*p* = 0.77
Positive History of Mother’s Gestational Diabetes	1 (10%)	1 (14.30%)	*p* = 0.78
Positive Family History for Diabetes	9 (90%)	2 (28.60%)	Chi squared test: *p* = 0.009
Positive Family History for Autoimmunity	0 (0%)	1 (14.30%)	Fisher’s exact test: *p* = 0.41
Fasting Morning Glucose Concentrations in mg/dl (a) During Screening Process	104 (100–107.50)	110 (103–119)	*p* = 0.23
Fasting Morning Glucose Concentrations in mg/dl (b) During Screening Process	105.5 (98.25–110.25)	110 (103–118)	*p* = 0.60
OGTT Glucose 0′ in mg/dl	101 (95.25–103.75)	99 (95–119)	*p* = 0.62
OGTT Glucose 30′ in mg/dl	147.50 (127.25–164.50)	160 (126–191)	*p* = 0.52
OGTT Glucose 60′ in mg/dl	109 (88.75–166.25)	148 (138.5–195)	*p* = 0.22
OGTT Glucose 90′ in mg/dl	107 (90–139)	128 (111–158.50)	*p* = 0.26
OGTT Glucose 120′in mg/dl	90 (82–123)	116 (89.50–123)	*p* = 0.53
OGTT Insulin 0′ in pmol/L	10.50 (4.20–17.20)	20.7 (12.10–25.40)	*p* = 0.07
OGTT Insulin 30′ in pmol/L	45.80 (37.80–100)	106 (34.65–215)	*p* = 0.43
OGTT Insulin 60′ in pmol/L	57.30 (25.10–73.40)	111 (70–178.50)	*p* = 0.10
OGTT Insulin 90′ in pmol/L	45.10 (20.90–86.7)	100 (61.2–146.90)	*p* = 0.14
OGTT Insulin 120′ in pmol/L	46.80 (23.40–74)	84 (39.10–107.50)	*p* = 0.26
Abbreviations: OGTT: Oral Glucose Tolerance Test			

**Table 2:** Differences in glycemic control, gut digestion, absorption, immunity, and metabolism measures before and after the intervention.

Variable	Control Group *N* = 10 (Median-IQR)	Intervention Group *N* = 7 (Median-IQR)	*p* Statistical Significance Mann–Whitney Test
**HbA1c in percentage (%) ^†^ and Fasting Blood Glucose Concentrations in mg/dl**			
Baseline	5.10 (5–5.25)	5.2 (5–5.5)	*p* = 0.74
Post-Intervention	5 (4.95–5.125)	5 (4.8–5.3) *	*p* = 0.96
**Morning Fasting Glucose**			
Baseline	102 (100–108.25)	108 (105–109) *	*p* = 0.06
1st month	111.5 (105.25– 119)	99 (94–108) *	*p* = 0.04
2nd month	102.50 (99.25–104.50)	103 (93.25–104.25) *	*p* =0 1
3rd month	104.50 (100–107.50)	98 (88–105) *	*p* = 0.33
4th month	108.50(100.50–113)	102 (90–113) *	*p* = 0.33
**Differences in Gut Digestion and Absorption Markers**			
**Total Fecal Fat (Valerate, Isobutyrate, Isovalerate) in mg/g**			
Baseline	28.70 (17.32–35.65)	10.90 (4.65–21.30)	*p* = 0.03
Post-Intervention	17.65 (14.67–34.10)	22.15 (6.62–32.27)	*p* = 0.762
**Triglycerides in mg/g**			
Baseline	1.55 (0.45–2.75)	0.70 (0.25–1.15)	*p* = 0.22
Post-Intervention	22.15 (6.62–32.27)	1.40 (0.5–6.42)	*p* = 0.61
**Long-Chain Fatty Acids in mg/g**			
Baseline	17.40 (11.05–24.50)	5.10 (2.90–13.25)	*p* = 0.03
Post-Intervention	11.10 (6.62–21)	10.30 (3.20–18.97)	*p* = 0.91
**Cholesterol in mg/g**			
Baseline	1.30 (0.97–2.55)	1.50 (0.65–4.5)	*p* = 0.72
Post-Intervention	1.60 (0.82–2.60)	1.30 (0.55–3.17)	*p* = 0.76
**Phospholipids in mg/g**			
Baseline	5.40 (3.75–9.90)	1 (0.75–3.85)	*p* = 0.01
Post-Intervention	4.45 (1.92–11.22)	4.95 (1.70–8.57)	*p* = 0.91
**Gut Immunology and Inflammation measures**			
**Calprotectin in mcg/g**			
Baseline	53.50 (18–111.25)	17 (16–41)	*p* = 0.12
Post-Intervention	17.50 (16–32.25) *	17 (17)	*p* = 0.76
**Eosinophil Protein X (EPX) in mcg/g**			
Baseline	7 (7)	0.70 (0.60–4.15)	*p* = 0.09
Post-Intervention	2.55 (0.40–4.60)	0.65 (0.45–1)	*p* = 0.76
**Gut Metabolism Markers**			
**n-Butyrate Concentration in micromole/g**			
Baseline	17.75 (10.55–25.75)	14.20 (7.50–29.25)	*p* = 0.88
Post-Intervention	17.05 (15.85–18.35)	20.30 (8.22–31.40)	*p* =0 1

^†^ DCCT (Diabetes Control and Complications Trial) units * Observed *p* ≤ 0.05 either in Wilcoxon’s or Friedman’s test.

**Table 3:** Group differences in intestinal microbiome populations at baseline and post-intervention.

	Control Group *N* = 10 Median (IQR)	Intervention Group *N* = 7 Median (IQR)	*p* Statistical Significance Mann–Whitney Test
***Prevotella* spp.**			
Baseline	8.9 × 10^6^ (5.75 × 10^6^ –1.45 × 10^7^ )	2.5 × 10^6^ (6.6 × 10^5^ –9.1 × 10^6^)	*p* = 0.16
Post-Intervention	8.35 × 10^6^ (5.4 × 10^6^–2.12 × 10^7^)	1.1 × 10^6^ (8.25 × 10^5^–1.07 × 10^7^)	*p* = 0.08
***Barnesiella* spp.**			
Baseline	1.6 × 10^8^ (1.14 × 10^8^–1.82 × 10^8^	3.8 × 10^7^ (3.3 × 10^6^–2.15 × 10^8^)	*p* = 0.10
Post-Intervention	2.15 × 10^8^ (1.6 10^8^–5.87 × 10^8^)	1.7 × 10^7^ (3.5 × 10^6^–2.07 × 10^7^)	*p* = 0.01
***Anaerotruncus colihominis***			
Baseline	4.8 × 10^6^ (1.72 × 10^6^–1.7 × 10^7^)	5.1 × 10^6^ (2.9 × 10^6^–6.8 × 10^6^)	*p* = 0.71
Post-Intervention	1.4 × 10^7^ (4.92 × 10^6^–2.35 × 10^7^)	3.4 × 10^6^ (1.04 × 10^6^–8.32 × 10^6^)	*P* = 0.06
***Butyrivibrio crossotus***			
Baseline	1.9 × 10^4^ (8.35 × 10^3^–5.9 × 10^4^)	1 × 10^4^ (6.35 × 10^3^–4.9 × 10^4^)	*p* = 0.06
Post-Intervention	1.9 × 10^5^ (7.87 × 10^4^–5 × 10^5^) *	2.45 × 10^4^ (9.7 × 10^3^–3 × 10^4^)	*p* = 0.01
***Faecalibacterium prausnitzii***			
Baseline	7.05 × 10^9^ (2.97 × 10^9^–1.175 × 10^10^)	4.3 × 10^9^ (2.25 × 10^9^–6 × 10^9^)	*p* = 0.16
Post-Intervention	8.55 × 10^9^ (6.8 × 10^9^–1.1 × 10^10^)	2.65 × 10^9^ (9.17 × 10^8^–5.12 × 10^9^)	*p* = 0.01
***Collinsella aerofaciens***			
Baseline	6.4 × 10^8^ (9.65 × 10^7^–8.05 × 10^8^)	2.7 × 10^8^ (8.5 × 10^6^–8.75 × 10^8^)	*p* = 0.50
Post-Intervention	9 × 10^8^ (2.035 × 10^8^– 1.45 × 10^9^)	1.4 × 10^7^ (1 × 10^4^–4.64 × 10^8^)	*p* = 0.03
***Escherichia coli***			
Baseline	2.7 × 10^7^ (5.02 × 10^6^–4.25 × 10^7^)	3.8 × 10^7^ (1.61 × 10^7^–4.65 × 10^7^)	*p* = 0.37
Post-Intervention	4.4 × 10^7^ (2.4 × 10^7^–7.5 × 10^7^)	5.65 × 10^6^ (1.97 × 10^6^–1.30 × 10^7^)	*p* = 0.01
***Methanobrevibacter smithii***			
Baseline	3.5 × 10^7^ (9.37 × 10^5^–8.675 × 10^7^)	5.8 × 10^7^ (7.9 × 10^5^–8.6 × 10 ^7^)	*p* = 0.88
Post-Intervention	8.6 × 10^7^ (6.45 × 10^7^–1.25 × 10^8^)	2.9 × 10^7^ (1 × 10^4^–7.9 × 10^7^)	*p* = 0.09
***Fusobacterium* spp.**			
Baseline	6.1 × 10^4^ (5.3 × 10^4^–1.475 × 10^5^)	3.7 × 10^4^ (1.7 × 10^4^–9 × 10^4^)	*p* = 0.07
Post-Intervention	1.35 × 10^5^ (3.605 × 10^4^–2.225 × 10^5^)	7.6 × 10^3^ (2.42 × 10^3^–9.25 × 10^4^)	*p* = 0.08
***PROTEOBACTERIA* PHYLUM**			
Baseline	3.1 × 10^7^ (2.5 × 10^7^–5.9 × 10^7^)	4.7 × 10^7^ (1.7 × 10^7^–6.4 × 10^7^)	*p* = 1
Post-Intervention	6.5 × 10^7^ (2.8 × 10^7^–1.4 × 10^8^)	2.2 × 10^7^(6.5 × 10^6^–4 × 10^7^)	*p =* 0.08
***Akkermansia muciniphila***			
Baseline	7.6 × 10^6^ (6.52 × 10^5^–1.55 × 10^7^)	6.5 × 10^5^ (1 × 10^4^–1.015 × 10^6^)	*p* = 0.06
Post-Intervention	2.8 × 10^6^ (1.2 × 10^6^–1.675 × 10^7^) *	5.05 × 10^5^ (1 × 10^4^–2.275 × 10^6^)	*p* = 0.03

* Observed *p* ≤ 0.05 either in Wilcoxon’s or in Friedman’s test.

**Table 4:** Participants’ compliance with probiotic supplementation (consumption of probiotics sachets and respective percentages of sachets consumed out of the number that should be consumed).

Month: Mean (± SD) consumed probiotics sachets/number of sachets that should be consumed
1st: 38.00 (± 21.48)/56.00 (67%)
2nd: 28.00 (± 16.52)/56.00 (50%)
3rd: 30.86 (± 16.3)/56.00 (55%)
4th: 31.43 (± 20.53)/56.00 (56%)
